# Silicone Wristbands as Passive Samplers in Honey Bee Hives

**DOI:** 10.3390/vetsci7030086

**Published:** 2020-07-06

**Authors:** Emma J. Bullock, Alexis M. Schafsnitz, Chloe H. Wang, Robert L. Broadrup, Anthony Macherone, Christopher Mayack, Helen K. White

**Affiliations:** 1 Department of Chemistry, Haverford College, 370 Lancaster Ave, Haverford, PA 19041, USA; ejbvt15@gmail.com (E.J.B.); amschafsnitz@gmail.com (A.M.S.); chloe.wang18@gmail.com (C.H.W.); rbroadru@haverford.edu (R.L.B.); 2 Life Science and Chemical Analysis Group, Agilent Technologies, 5301 Stevens Creek Blvd, Santa Clara, CA 95051, USA; anthony_macherone@agilent.com; 3 Department of Biological Chemistry, The Johns Hopkins University School of Medicine, 733 N Broadway, Baltimore, MD 21205, USA; 4 Department of Biology, Swarthmore College, 500 College Ave, Swarthmore, PA 19081, USA; cmayack@sabanciuniv.edu; 5 Molecular Biology, Genetics, and Bioengineering, Faculty of Engineering and Natural Sciences, Sabancı University, 34956 Istanbul, Turkey

**Keywords:** *Apis mellifera*, bee, silicone band, hive, passive sampler

## Abstract

The recent decline of European honey bees (*Apis mellifera*) has prompted a surge in research into their chemical environment, including chemicals produced by bees, as well as chemicals produced by plants and derived from human activity that bees also interact with. This study sought to develop a novel approach to passively sampling honey bee hives using silicone wristbands. Wristbands placed in hives for 24 h captured various compounds, including long-chain hydrocarbons, fatty acids, fatty alcohols, sugars, and sterols with wide ranging octanol–water partition coefficients (K_ow_) that varied by up to 19 orders of magnitude. Most of the compounds identified from the wristbands are known to be produced by bees or plants. This study indicates that silicone wristbands provide a simple, affordable, and passive method for sampling the chemical environment of honey bees.

## 1. Introduction

The dwindling global population of the honey bee pollinator *Apis mellifera*, the main pollinator species used in agriculture, has driven researchers to investigate honey bees and their responses to different environmental and pathological conditions [1,2]. These insects play a vital role in agricultural systems and almost a third of the global food crop depends exclusively on the European *Apis mellifera* species [3]. Since the 1940s, the number of managed honey bee colonies in the United States has declined, with the most dramatic losses occurring in the past decade [4]. Numerous factors have been implicated in the decline in bee health including exposure to pesticides and viruses [5], the spread of *Varroa destructor* mites [6], and parasites such as *Nosema ceranae* [7]; however, none can be identified as a single underlying causal factor.

Due to the vast number of variables and interactions that impact honey bee health, identifying the cause, impact, and risk associated with individual factors is challenging. Targeted exposure studies assessing chemicals and contaminants including pesticides and pathogens have been examined [6]. Recently, an exposomic approach has explored a more comprehensive approach where external chemicals as well as those produced by honey bees are combined into a single analysis [8]. Some compounds produced by bees, known as semiochemicals, are released by individual honey bees as cues or signals in order to relay information to hive mates, allowing honey bees to coordinate their responses to environmental stimuli as a hive [9]. Semiochemicals can also ward off or attract other honey bees [9], can relay the needs of larvae to nurse bees [10,11], and are involved in social recognition [12,13]. Certain chemicals have the potential to relay specific information about the disease state of an individual. For example, ethyl oleate is a chemical produced by honey bees that is significantly increased by infection of the gut fungus *N. ceranae* [13], and cuticular hydrocarbon profiles of honey bees have been observed to change when exposed to bacterial or *N. ceranae* infections [14,15]. It has also been shown that poor nutrition can lead larvae to release E-β-ocimene into the hive [11]. Monitoring chemicals produced by honey bees is of significant interest as it can provide insight into specific infection status, pesticide exposure, as well as nutrition deficiencies, thus providing insight into the overall health of a bee colony.

Semiochemicals and other volatile and semivolatile chemicals (VOCs and SVOCs) found in honey bee hives have previously been sampled by collecting the air in the hive directly via a syringe and then examining the chemical contents of the air sample via gas chromatography–mass spectrometry (GC–MS) [16]; however, this method does not allow the storage of samples prior to analysis [16]. Alternative methods have utilized adsorbent packing materials (such as Super Q, Hayesep Q, Porapak Q, Tenax TA) combined with vacuum/air tube systems [10,16,17]. These methods have limitations as the vacuum/air tube systems are complicated to construct and their presence may cause vibrations that induce a stress response in the bees and bias the chemical profiles measured [16]. Solid-phase microextraction (SPME) fibers have been used as passive samplers, avoiding the problems involved in complicated air flow setups; however, they are somewhat limited as they are designed to target specific chemicals, and are expensive and fragile to use [16,17,18,19,20,21].

This study aims to apply silicone wristbands, a previously established method [22,23,24,25,26], as a new approach to passively sample a wide range of chemicals present in honey bee hives. Silicone wristbands are cheap, commercially available, and have been used as passive environmental samplers to provide time-averaged concentrations of human exposures to polycyclic aromatic hydrocarbons (PAHs), consumer products, personal care products, pesticides, phthalates, other industrial compounds [22,23,24], and organophosphate flame retardants [25,26]. Compounds adsorbed by silicone wristbands have been found to remain on the bands for extended periods of time, unlike compounds on SPME fibers [24,25,26,27]. Perhaps the most significant advantage of silicone wristbands is that they are able to sample VOCs and SVOCs with octanol-air partition coefficients (K_oa_) ranging in 10 orders of magnitude [22,23,24]. The K_oa_ as well as the octanol–water partition coefficients (K_oaw_) of a chemical compound are values that represent the hydrophobicity of a compound. Specifically, these values describe the ratio of a compound in octanol divided by the concentration of the same compound in air or water for K_oa_ and K_ow_, respectively. With respect to passive samplers, these values are used to show the range in hydrophobicity of compounds that a sampler can detect, which, as mentioned, is large for silicone wristbands. Their ability to adsorb such a broad range of compounds makes them ideal for sampling chemicals produced by bees and could potentially collect a broader range of compounds than the aforementioned SPME fibers and polymer samplers.

In this study, commercially available silicone wristbands were pre-cleaned and placed in honey bee hives to sample VOCs and SVOCs. This study presents information about the types of chemicals that can be adsorbed and analyzed to provide a reliable chemical profile of honey bee hives from a variety of urban and suburban locations.

## 2. Materials and Methods

### 2.1. Materials

All solvents were GC Resolv or Optima grade, obtained from Fisher Scientific (Newark, NJ, USA). Glassware was combusted at 450 °C for 8 h before use. 

### 2.2. Band Preparation 

Silicone bands (acquired from 24HourWristbands.com) with a circumference of 20 cm, ≈40 cm^2^ surface area, and ≈4 g of sorbent were prepared according to a modified procedure [22]. Briefly, bands were cleaned in previously combusted (450 °C, 8 h) glass jars with a 1:1 volume mixture of ethyl acetate (EtOAc) to hexanes and shaken at 80 rpm for at least 2.5 h before the removal of the solvent. This procedure was repeated three more times, at which point the bands were removed from the jar and placed on combusted aluminum foil in a high-vacuum pressure oven at 60 °C for 48 h. The bands were stored individually in 40 mL combusted amber glass vials at room temperature prior to use. 

### 2.3. Band Deployment 

**Figure 1:**
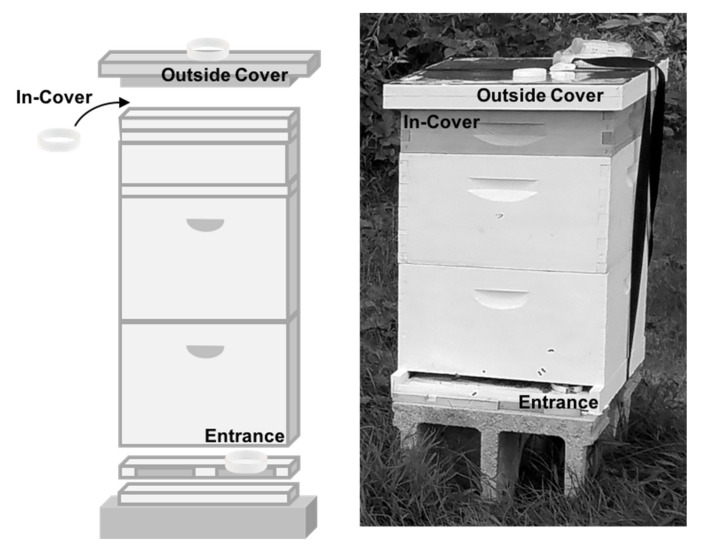
Diagram of a hive (**left**) and photo of a hive in the field (**right**), highlighting the placement of bands. In-cover band for the hive in the field is not shown as it is inside the cover part of the hive. Wristbands shown are 20 cm in circumference and 1 cm width; hives are approximately 1 × 0.5 × 0.4 m.

During July, August, September, and October of 2016, bands were deployed in 10 apiaries in rural, suburban, and urban areas of southeastern Pennsylvania (details provided in Table S1 of the Electronic Supplementary Materials). At each apiary, three hives were sampled across three time points. In order to capture different parts of the environment inhabited by the honey bees, we placed one silicone band under the top of each hive (in-cover) and placed one inside the entrance of each hive. An additional band was placed on top of each hive (outside cover) to serve as an external control (details shown in Figure 1). The bands were retrieved 24 h later and transferred to individual 40 mL amber vials, placed on dry ice, transported back to the lab, and stored at −20 °C. 

### 2.4. Band Extraction 

Silicone bands were cleaned and extracted according to a modified procedure [22]. Briefly, each band was rinsed with Milli-Q water to remove any solids, such as dirt, propolis, or stingers, followed by isopropyl alcohol to remove water. The bands were then placed in individual 500 mL jars with 100 mL EtOAc and shaken at 60 rpm for 2 h. The extract was removed and the extraction process was repeated twice more before the extracts were combined, reduced in volume via rotary evaporation to approximately 1 mL, and stored at −20 °C prior to further analysis. The extracts were then solvent-exchanged into hexane, spiked with perdeuterated *n*-hexadecane as an internal standard (average recoveries were 74%), and charged onto a small glass pipette column (0.5 cm × 6 cm) packed with fully activated silica gel (100–200 mesh). The first fraction (F1) was eluted from the column with 4mL of hexane, followed by the second fraction (F2) eluted with 4 mL of an equal mixture of dichloromethane and methanol containing 1% formic acid. Laboratory blanks run alongside samples contained no detectable compounds.

### 2.5. Gas Chromatographic Analysis of Band Extracts

The F1 extracts were analyzed on a 1D Agilent 7890 series gas chromatograph coupled to a flame ionization detector (FID, Santa Clara, CA, USA). Compounds were separated on a J&W DB-XLB capillary column (30 m, 0.25 mm I.D., 0.25 μm film) with helium carrier gas at a constant flow of 1 mL min^−1^. The GC oven had an initial temperature of 40 °C (1 min hold) and was ramped at 10 °C min^−1^ until 160 °C (1 min hold), then ramped again at 4 °C min^−1^ until 320 °C (36 min hold). Quantities of *n*-alkanes were calculated using response factors determined from pure standards. Presence of *n*-alkenes in the F1 extracts were confirmed by analyzing select extracts on an Agilent 7890 series gas chromatograph with an Agilent 5975 mass selective detector (MSD) and were noted, though not quantified. All fractions from each in-cover, outside, and entrance band for 10 hives were analyzed via GC–MS with a compound detection limit of 0.1 ng/µL determined from a standard curve using pure standards.

In order to identify unknown compounds, we analyzed F2 extracts via a 1D Agilent 7890 series gas chromatograph coupled to an Agilent 5975 mass selective detector (MSD). Prior to analysis, F2 extracts were derivatized with bis(trimethylsilyl)trifluoroacetamide (BSTFA) in pyridine. Compounds were separated on a J&W DB-XLB capillary column (60 m, 0.25 mm internal diameter (I.D.), 0.25 μm film) with helium carrier gas at a constant flow of 1 mL min^−1^. The GC oven had an initial temperature of 40 °C (1 min hold) and was ramped at 10 °C min^−1^ until 160 °C (1 min hold), then ramped again at 4 °C min^−1^ until 320 °C (36 min hold). The MS was operated in electron-impact (EI) mode with an ionization energy of 70 eV. Spectra were acquired between *m*/*z* 40–650 at a scan rate of 1 cycle s^−1^. Fatty acids and fatty alcohols were identified from mass spectral and retention time characteristics compared to pure standards. All other compounds were tentatively identified from mass spectra and gas chromatographic retention characteristics.

## 3. Results

### 3.1. Chemical Profiles of Hive Air Were Dominated by Hydrocarbon Compounds

**Figure 2:**
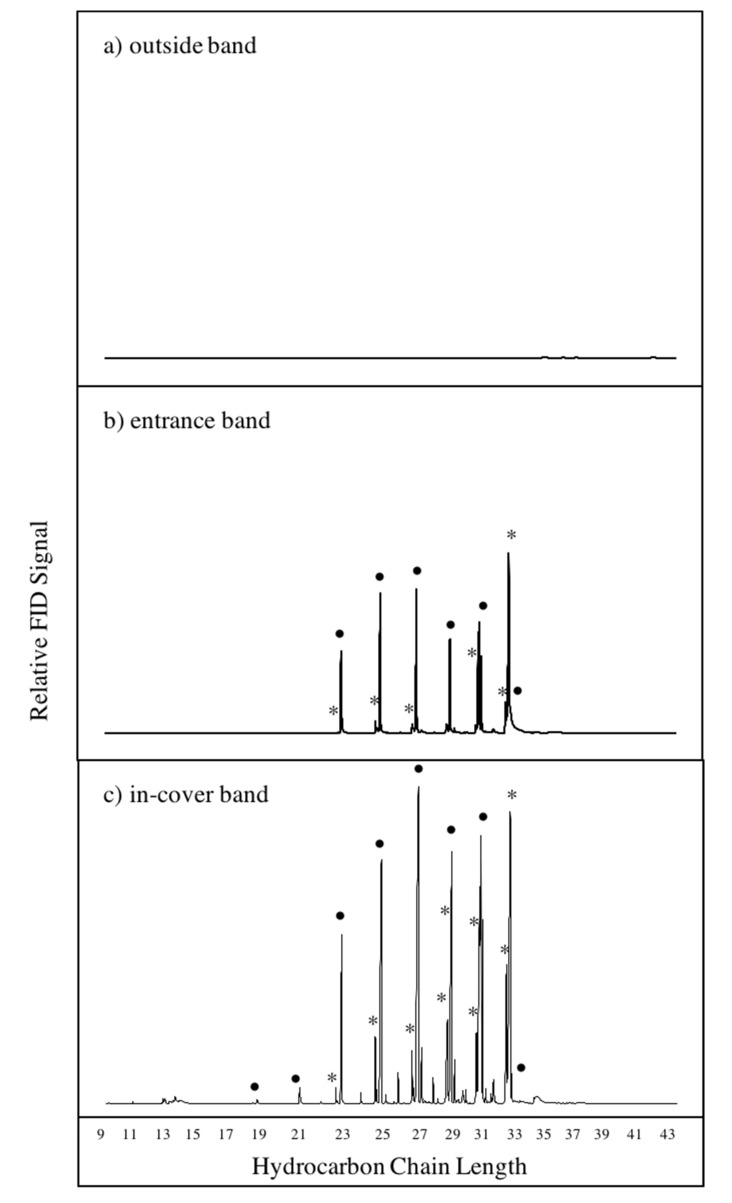
Representative gas chromatograms of compounds extracted from (**a**) an outside band, (**b**) an entrance band, and (**c**) an in-cover band. Alkanes indicated with dots (•) and alkenes indicated with asterisks (*). All bands were deployed in the same hive at Awbury Arboretum on 07/15/2016.

**Figure 3:**
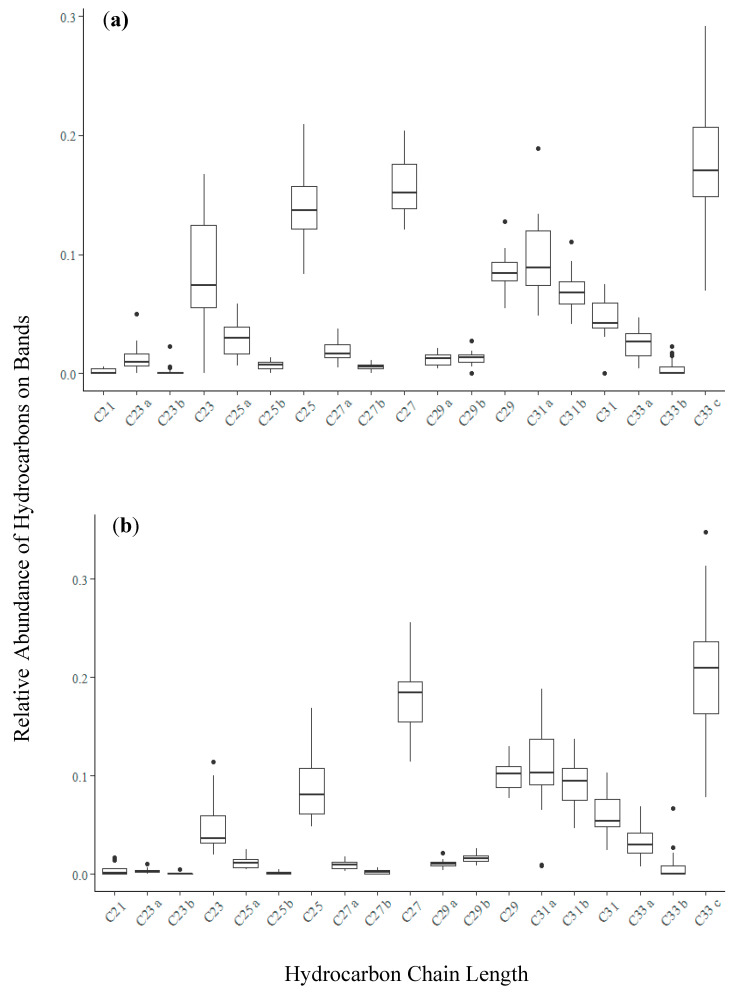
Box and whisker plots illustrating the relative abundance of hydrocarbons for all samples collected from (**a**) the entrance and (**b**) in-cover of hives. Boxes contain the interquartile range—50% of data—with center line indicating the median value of data. Whiskers indicate minimum and maximum values within 1.5 times above the 75th percentile or below the 25th percentile, with dots representing outliers.

Bands placed in-cover and at the entrance of hives exhibited similar chemical profiles dominated by *n*-alkanes and *n*-alkenes with odd chain lengths between C_21_–C_33_ (Figure 2 and Figure 3). The chemical profiles of *n*-alkanes and *n*-alkenes for the entrance bands had more variability between hives (Figure 3), likely due to the fact that entrance bands come into more frequent physical contact with the honey bees than the bands placed in-cover. Bands placed on the outside of hives did not contain compounds at detectable levels (Figure 2), and thus acted as field controls in this study. Differences in the chemical profiles from bands placed in hives at different locations were observed (Figure 3), but were not significant between the location types (rural, suburban, and urban) examined. For the remainder of this study, we chose to focus on the in-cover bands, which have similar chemical profiles to the entrance bands but represent the chemical profile of the hive air with less frequent physical contact, and are practically easier to recover. 

### 3.2. Bands Adsorbed Bee-Associated Chemicals 

The predominant compounds detected from the bands (*n*- alkanes and *n*-alkenes with odd chain lengths between C_21_–C_33_) are honey bee semiochemicals (Table 1), some of which are known nestmate recognition semiochemicals [28,29,30]. Four of these compounds—C_23_- and C_25_-*n*-alkane, and C_23_- and C_25_-*n*-alkene—are also known to be vital for the waggle dance, which relays information about the whereabouts of food [9,31]. Oleic acid, linoleic acid, and α-linolenic acid were also detected in at least half of the samples. These compounds are known to be major constituents of pollen [32] and beeswax [33], and have been detected in worker bees [34,35]. Certain saturated fatty acids and fatty alcohols, previously detected in worker bees [34], drone cocoons [36], or as a constituent of queen retinue pheromone [31], were identified broadly across samples. The alarm pheromone, [Z]-11-eicosenol, which is known to be released by bees, was also observed [31,34]. Glycerol was found in 78% of samples, and is known to participate in the ester biosynthetic pathway in bees [31]. Chrysin is a compound found in honey and was only observed in 13% of samples [37].

### 3.3. Bands Adsorb Plant-Derived Compounds

Plant-derived compounds were also extracted from bands, as well as the fatty acid C9:0, a common non-selective herbicide (Table 2) [41]. Fatty acids with carbon ranges between C10:0–C22:0 are known to be derived from plants and have been identified in pollen, along with oleic, linoleic, and α-linolenic acid, as previously described [32]. Further, the fatty alcohol 1-tritriacontanol, which has been shown to have a plant origin, was detected [42,43]. Plant-originating allelochemicals were also detected, including benzoic acid and cinnamic acid derivatives [44,45]. In fewer samples, sterols and sugars were observed, likely to originate from pollen and nectar, respectively [46,47]. All compounds identified from bands placed in hives are described in Table S2 of the Supplementary Materials.

## 4. Discussion

### 4.1. Implications for Studies of Honey Bee Health

In this approach of using silicone wristbands to passively sample chemicals present in honey bee hives, we found that all but one of the chemicals detected were associated with bees and plants as opposed to human activity or viruses. If these types of compounds were present, our inability to detect them likely arises from them being present at lower abundances, giving a smaller signal that is either masked by the much larger signal of the other abundant compounds present, or that it is outside of our detection limits. Our approach, however, is still of use, as several of the compounds detected on the bands have been implicated in studies concerning honey bee health and nutrition.

Compounds related to honey bee health include those produced by bees such as octadecanoic acid and (9Z)-octadecenoic acid. These two compounds have been observed to be produced more in hives that have large numbers of bees infected with *N. ceranae*, possibly as precursors to short fatty acid chains known to have antibiotic properties [8]. Hexadecanoic acid is a known regulator of fatty acid synthesis in bees and has been observed in lower quantities when a hive is infected [8]. Tricosanoic acid and tetracosanoic acid were both detected on bands, and while not produced by honey bees, are known *Varroa destructor* mite semiochemicals [35]. The alkane tricosane has also been shown to be produced more by bees upon infection by Gram-negative bacteria [14]. Overall, it is known that infection from various pathogens causes honey bees to alter their hydrocarbon profiles [15], which could be examined in future studies by analyzing the chemical profiles of bands placed in hives.

The plant-derived compounds identified in this study could also be used to extrapolate information about hive health, as honey bees obtain all necessary proteins, lipids, and vitamins from pollen [32]. It has been shown that not only the quantity of pollen collected, but also the quality and diversity of compounds available in the pollen, determine hive productivity and longevity [32]. As a result, the detection of numerous compounds from contact with pollen could help researchers investigate the impact of local flora on honey bee hives. These results indicate that deploying silicone bands in honey bee hives may help researchers gain insight into several different aspects of honey bee health, including the regulation of important metabolic pathways, the presence of parasites and pathogens, and the quality of pollen being gathered.

### 4.2. General Applicability of Bands to Honey Bee Research

The log octanol–water partition coefficients (Log K_ow_) for compounds in this study range from −3.38 to 16.6, as reported in the Hazardous Substances Data Bank (HSDB) [40]. This exceeds the capacity of most passive samplers currently in use [24], overcoming the major drawback of currently used passive sampling methods in hives that target certain compound groups [16,17,18,19]. The diversity in compounds adsorbed by silicone bands thus removes the necessity for multiple passive samplers when the compounds of interest vary significantly in structure and physical and chemical properties. Despite our expectation that this method would primarily target VOCs and SVOCs, non-volatile compounds were consistently seen across samples, arising from direct contact of the bees with the bands. If future implementation of silicone bands in hives chose to sample VOCs and SVOCs only, then an approach to maintain separation between the bees and bands would be needed (e.g., fine wire mesh cage for the bands).

This study also provides a method that allows researchers to quantitatively compare hive chemical profiles without needing to determine compound concentrations in the air of each hive. Previous studies have shown that the consistent adsorption of silicone bands allow samples to be compared without the need for further calculations, provided the dimensions of the silicone sampler and deployment length are the same [25,48]. Silicone bands are more consistent in their compound adsorption ratios than several other passive samplers such as polyurethane foam, urine sampling, and hand wipes [25,26], potentially because the silicone matrix stabilizes compounds until extraction [24].

Unlike SPME fibers, silicone bands can be used for quantitative analysis of compounds, provided that any comparisons are between band samples, as concentrations adsorbed to the bands do not directly represent environmental concentrations [22,24,25]. It has also been shown that concentrations of volatile compounds adsorbed by the silicone matrix remain stable under transport conditions of 30 °C for 7 days and under storage conditions of −20 °C for approximately 6 months [24]. To compare concentrations of chemicals from different compound classes between bands, the specific affinities of the silicone matrix to different compounds would need to be examined. In addition, to connect the chemical compounds detected to the health of the hive, a future study would need to also measure specific infections and diseases, such as *N. ceranae*, the *Varroa* destructor mite, deformed wing virus, and European foulbrood, in addition to chemical compounds of interest. Any future study must be designed to be large enough so that statistical testing between chemicals and health to be made.

### 4.3. Modifications for Future Studies

One concern with the use of silicone wristbands is the large amount of solvent needed to clean and process the bands; however, in this study, the amount of sample necessary for compounds to be detected via GC–MS and GC–FID was approximately 3 × 10^−5^ times our original extract per run. This shows that future studies could use fractions of silicone wristbands, rather than the entire band, significantly reducing the amount of solvent needed per sample. Researchers would have to ensure that each section of band being deployed was the same length, width, thickness, and surface area if they wished to compare concentrations of compounds between samples. It would also be necessary to ensure each band is deployed for the exact same duration, as many of the compounds may not reach equilibrium and could exhibit time-dependent concentrations [24].

Since the bands are capable of adsorbing both volatile and non-volatile chemicals, the placement of the bands in the hive will be critical to ensure non-biased quantitative analysis. If researchers are interested in volatile compounds, isolating the bands from the bees using a mesh cage (as previously mentioned) will be essential to obtain non-biased results. If the researcher is interested in non-volatile compounds, such as hydrocarbons or pesticides, they could pin the band flat in front of the entrance of the hive, requiring bees to walk over it when entering and exiting the hive. This approach would make the quantification of chemicals challenging, but it would provide a broad overview of the compounds worker bees are carrying into the hive.

## 5. Conclusions

Considering the ease with which chemicals in bands can be compared, the minimal disturbance to the hive, and the variety of compounds detectable, using silicone bands to investigate the relationship between chemical compounds and honey bees shows great potential. Further, our results show that bands did not collect detectable compounds from outside of the hive, as no compounds were detected on the outside bands. This contrasts with SPME fibers, which are easily contaminated by background volatiles [16] and are quite expensive. Researchers can use bands as samplers in the open hive environment, as was done in this study, as well as in closed sampling containers. In a closed system, it would be possible to sample the volatile chemicals released by bees or adhered to the surface of bees based on certain castes, age groups, or environmental conditions, without the need for complicated air flow systems or filters. As a result, we believe using silicone band passive samplers provides alternative, flexible, more affordable opportunities to explore the chemical ecology of honey bees and the factors that influence their health, behavior, and survival.

## Figures and Tables

**Table 1:** The percentage of silicone bands placed in honey bee hives that contain honey bee semiochemicals and honey bee-associated compounds organized by compound type.

Compound Type	Chain Lengths	Log K_ow_ Range	% of Bands ^a^	Association with Honey Bees
Alkanes	C_21_–C_33_ odd chain lengths only	10.7–16.6 ^b^	44–95	nestmate recognition semiochemical [15,28,29,30,38]
				queen tergal gland secretion [38]
				waggle dance (C_23_ and C_25_ only) [9,31]
Alkenes	C_23_–C_33_^b^ odd chain lengths only	11.4–16.4 ^b^	7–93	nestmate recognition semiochemical [29,30,31,33]
				queen tergal gland secretion [38]
				waggle dance (C_23_ and C_25_ only) [9,31]
Fatty acids	C12:0–C30:0	3.42–13.8	53–97	detected in worker bees (C12:0–C22:0; C26:0–C30:0 even chain lengths) [34,35]
				detected in *Varroa* destructor (C23:0–C24:0) [35]
Unsaturated fatty acids	C18:1 (oleic acid)	7.64	96	major constituent—beeswax [35] detected in worker bees [34,35]
	C18:2 (linoleic acid)	7.05	81	major constituent—beeswax [31]
				detected in worker bees [34,35]
	C18:3 (α-linolenic acid)	6.46	48	major constituent—beeswax [31]
				detected in worker bees [34,35]
Fatty alcohols	C_16_–C_32_	6.83–14.10 ^b^	36–96	queen retinue pheromone (QRP) (C_16_) [31]
				drone cocoon (C_17_) [36]
				detected in worker bees (C_18_–C_32_) [34]
	C_19_^c^		40	detected in *Bombus ruderarius* and *Bombus sylvarum* (Hymenoptera, Apidae) [39]
	C_19_^c,d^		76	detected in *Bombus ruderarius* and *B. sylvarum* (Hymenoptera, Apidae) [39]
	C_20_^c^ [Z]-11-eicosenol		76	alarm pheromone [31,34]
Other	Chrysin	3.52	13	honey, propolis, and beeswax [37]
	Glycerol	-1.76	78	ester biosynthesis in honey bees [31]

^a^ Specific percentages are for individual compounds. A range of values corresponds to the range of compounds described. ^b^ Log K_ow_ values estimated using the Crippen method EPISuite KOWWIN v1.67 estimate (USEPA) (HSDB [40]). ^c^ Alkenes identified by molecular weight and fragmentation patterns. Exact location of the double bonds is unknown. ^d^ Alkenes with two double bonds.

**Table 2:** The percentage of silicone bands placed in honey bee hives that contain plant-associated compounds organized by compound type.

Compound Group	Compounds	Log K_ow_ Range^26^	% of Bands ^a^	Associations with Plants
Fatty acids	C10:0–C20:0; C22:0	4.0–9.9	64–97	pollen [32]
	C9:0	3.42	84	nonselective herbicide [41]
Unsaturated fatty acids	C18:1 (oleic acid)	7.64	96	
	C18:2 (linoleic acid)	7.05	81	all major constituents of pollen [32]
	C18:3 (α-linolenic acid)	6.46	48	
Fatty alcohols	C_33_		13	plant origin [42,43]
Bnzoic and cinnamic acid derivatives	benzoic acid	1.87	79	
	cinnamic acid, p-methoxy	2.68	63	
	cinnamyl cinnamate	3.96	12	plant originated allelochemicals [44,45]
	4-hydroxybenzoic acid	1.58	12	
	hydrocinnamic acid	1.84	10	
	benzyl cinnamate	3.44	9	
	ferulic acid	1.51	7	
	cinnamic acid, 3,4-dihydroxy-	1.15	3	
	benzyl salicylate	4.31 ^b^	3	
Sterols	beta-sitosterol (29Δ (5))	9.65 ^b^	43	pollen [46]
	stigmasterol (29Δ (5, 22))	9.43	13	pollen [46]
	lanosta-8,24-dien-3-ol, acetate, (2, β)-	11.8 ^b^	9	pollen [46]
Sugars	d-mannose	−3.38 ^b^	9	nectar [47]
	d-glucose	−2.82	6	nectar [47]
	d-glucopyranose	−2.82	3	nectar [47]
	d-xylose	−2.74 ^b^	3	nectar [47]

^a^ Specific percentages are for individual compounds. A range of values corresponds to the range of compounds described. ^b^ Log K_ow_ values estimated using the Crippen method EPISuite KOWWIN v1.67 estimate (USEPA) (HSDB [40]).

## Supplementary Materials

The following are available online at https://www.mdpi.com/2306-7381/7/3/86/s1, Table S1: Locations of hives sampled in this study, Table S2: All compounds identified on bands.
