# Supplementary material for: Silicone Wristbands as Passive Samplers in Honey Bee Hives
Source: Vet Sci. 2020 Jul 6;7(3):86. doi: 10.3390/vetsci7030086 (PMC7558201; doi:10.3390/vetsci7030086)
Supplement: Supplementary file 1 [file vetsci-07-00086-s001.pdf]

Supplementary Materials

Emma J. Bullock, Alexis M. Schafsnitz, Chloe H. Wang, Robert L. Broadrup, Anthony Macherone, Chris Mayack, Helen K. White

Table S1. Locations of hives sampled in this study.

Location	Description	Latitude	Longitude
Haverford College	suburban	40.0122° N	75.2996° W
Greensgrow Farms	urban	39.9785° N	75.1210° W
Awbury Arboretum	urban	40.0508° N	75.1681° W
private residence in Doylestown, PA	rural	40.2739° N	75.1169° W
Monastery of the Visitation Nuns	urban	39.9924° N	75.2434° W
Mt Moriah Cemetery	urban	39.9303° N	75.2338° W
private residence in Malvern, PA	suburban	40.0356° N	75.5157° W
Leapfrog Farm	rural	39.7859° N	75.9791° W
Swarthmore College	suburban	39.9007° N	75.3482° W
Temple University, Ambler Campus	rural	40.1662° N	75.2546° W

Table S2. All compounds identified on bands.

Groups	Compound Name	Chain Length	Log K_{ow}	CAS Number	No. of Bands	References
alkanes	*n*-heneicosane	C_{21}	10.7 [c]	629-94-7	36	nestmate recognition semiochemical [2 – 5] and queen tergal gland secretion [6]
	n-tricosane	C_{23}	11.6 [c]	638-67-5	73	waggle dance [7 – 8]
	n-pentacosane	C_{25}	12.6 [c]	629-99-2	73	waggle dance [7 – 8]
	n-heptacosane	C_{27}	13.6 [c]	593-49-7	76	nestmate recognition semiochemical [2 – 5] and queen tergal gland secretion [6]
	n-nonacosane	C_{29}	14.6 [c]	630-03-5	74	nestmate recognition semiochemical [2 – 5] and queen tergal gland secretion [6]
	n-hentriacontane	C_{31}	15.6 [c]	630-04-6	73	nestmate recognition semiochemical [2 – 5] and queen tergal gland secretion [6]
	n-tritriacontane	C_{33}	16.6 [c]	630-05-7	54	nestmate recognition semiochemical [2 – 5] and queen tergal gland secretion [6]
alkenes [a]	*n*-tricosene	C_{23}	11.4 [c]		35	waggle dance [7 – 8], nestmate recognition semiochemical [2 – 5], and queen tergal gland secretion [6]
	n-pentacosene	C_{25}	12.4 [c]		59	waggle dance [7 – 8], nestmate recognition semiochemical [2 – 5], and queen tergal gland secretion [6]
	n-pentacosene	C_{25}	12.4 [c]		11	waggle dance [7 – 8], nestmate recognition semiochemical [2 – 5], and queen tergal gland secretion [6]
	n-heptacosene	C_{27}	13.5		50	nestmate recognition semiochemical [2 – 5] and queen tergal gland secretion [6]
	n-heptacosene	C_{27}	13.5		13	nestmate recognition semiochemical [2 – 5] and queen tergal gland secretion [6]
	n-nonacosene	C_{29}	14.4 [c]		6	nestmate recognition semiochemical [2 – 5] and queen tergal gland secretion [6]
	n-nonacosene	C_{29}	14.4 [c]		54	nestmate recognition semiochemical [2 – 5] and queen tergal gland secretion [6]
	n-hentriacontene	C_{31}	15.4 [c]		73	nestmate recognition semiochemical [2 – 5] and queen tergal gland secretion [6]
	n-hentriacontene	C_{31}	15.4 [c]		71	nestmate recognition semiochemical [2 – 5] and queen tergal gland secretion [6]
	n-tritriacontene	C_{33}	16.4 [c]		54	nestmate recognition semiochemical [2 – 5] and queen tergal gland secretion [6]
	n-tritriacontene	C_{33}	16.4 [c]		75	nestmate recognition semiochemical [2 – 5] and queen tergal gland secretion [6]
	n-tritriacontene	C_{33}	16.4 [c]		54	nestmate recognition semiochemical [2 – 5] and queen tergal gland secretion [6]

	nonanoic acid	C9:0	3.4	112-05-0	56	nonselective herbicide [9]
	decanoic acid	C10:0	4.1	334-48-5	59	pollen [10]
	dodecanoic acid	C12:0	4.6	143-07-7	65	pollen [10]; detected in worker bees [11]
	tetradecanoic acid	C14:0	6.1	544-63-8	60	pollen [10]; detected in worker bees [11, 12]
	pentadecanoic acid	C15:0	6.5	1002-84-2	43	detected in varroa destructor [11]
	hexadecanoic acid	C16:0	7.2	57-10-3	68	major constituent – pollen [10]; beeswax [13]; detected in worker bees [11, 12]
	heptadecanoic acid	C17:0	7.5 [c]	506-12-7	59	detected in worker bees [11, 12] [12-13]
	octadecanoic acid [stearic acid]	C18:0	8.2	57-11-4	68	major constituent – pollen [10]; beeswax [13]; detected in worker bees [11, 12]
	oleic acid	C18:1	7.6	112-80-1	64	major constituent – pollen [10]; beeswax [13]; detected in worker bees [11, 12]
	linoleic acid	C18:2	7.1	60-33-3	54	major constituent – pollen [10]; beeswax [13]; detected in worker bees [11, 12]
fatty acids	α-linolenic acid	C18:3	6.5	463-40-1	32	major constituent – pollen [10]; beeswax [13]; detected in worker bees [11, 12]
	eicosanoic acid [arachidic acid]	C20:0	8.9 [c]	506-30-9	61	pollen [10]; detected in bee bread [11]
	heneicosanoic acid	C21:0	9.4 [c]	2363-71-5	52	detected in worker bees [11]
	docosanoic acid	C22:0	9.9 [c]	112-85-6	58	pollen [10]; detected in worker bees [11, 12]
	tricosanoic acid	C23:0	10.4 [c]	2433-96-7	58	detected in varroa destructor [11]
	tetracosanoic acid	C24:0	10.9 [c]	557-59-5	61	major constituent – beeswax [13]; detected in varroa destructor [11]
	pentacosanoic acid	C25:0	11.4 [c]	506-38-7	49	plant origin [14, 15]
	hexacosanoic acid	C26:0	11.9 [c]	506-46-7	53	detected in worker bees [12]
	octacosanoic acid	C28:0	12.9 [c]	506-48-9	55	detected in worker bees [12]
	triacontanoic acid	C30:0	13.8 [c]	506-50-3	44	detected in worker bees [12]
	1-hexadecanol	C_{16}	6.8 [c]	36653-82-4	36	queen retinue pheromone (QRP) [7]
	1-heptadecanol	C_{17}	7.2 [c]	1454-85-9	64	drone cocoon [16]
	1-octadecanol	C_{18}	7.7 [c]	112-92-5	62	detected in worker bees [12]; drone cocoon [16]
	1-nonadecanol	C_{19}	8.2 [c]	145-84-8	60	detected in worker bees [12]; drone cocoon [16]
	n-nonadecenol**	C_{19}			51	detected in bombus ruderarius and b. sylvarum (hymenoptera, apidae) [17]
	n-nonadecenol*	C_{19}			27	detected in bombus ruderarius and b. sylvarum (hymenoptera, apidae) [17]
	1-eicosanol	C_{20}	8.7 [c]	629-96-9	51	detected in worker bees [12]; drone cocoon [16]
	[z]-11-eicosenol	C_{20}	8.5 [c]	62442-62-0	51	alarm pheromone [7, 12]
	1-heneicosanol	C_{21}	9.2 [c]	15594-90-8	35	detected in worker bees [12]; drone cocoon [16]
fatty alcohols	1-docosanol	C_{22}	9.7 [c]	30303-65-2	45	detected in worker bees [12]; drone cocoon [16]
	1-tricosanol	C_{23}	10.2 [c]	3133-01-5	40	detected in worker bees [12]
	1-tetracosanol	C_{24}	10.7 [c]	506-51-4	57	detected in worker bees [12]
	1-pentacosanol	C_{25}	11.2 [c]	26040-98-2	54	detected in worker bees [12]
	1-hexacosanol	C_{26}	11.7 [c]	506-52-5	53	detected in worker bees [12]
	1-heptacosanol	C_{27}	12.1 [c]	2004-39-9	50	detected in worker bees [12]
	1-octacosanol	C_{28}	12.6 [c]	557-61-9	54	detected in worker bees [12]
	1-nonacosanol	C_{29}	13.1 [c]	6624-76-6	34	detected in worker bees [12]
	1-triacontanol	C_{30}	13.6 [c]	593-50-0	46	detected in worker bees [12]

	1-hentriacontanol	C_{31}	14.1 [c]	544-86-5	26	detected in worker bees [12]
	1-dotriacontanol	C_{32}		6624-79-9	24	detected in worker bees [12]
	1-tritriacontanol	C_{33}		71353-61-2	9	plant origin [18, 19]
other	glycerol		-1.8	56-81-5	63	ester biosynthesis in honey bees [7]
	benzoic acid		1.9	65-85-0	53	plant originated allelochemical [20, 21]
	cinnamyl alcohol		1.6	104-54-1	23	plant originated allelochemical [20, 21]
	trans-cinnamic acid		1.8 [c]	140-10-3	30	plant originated allelochemical [20, 21]
	hydrocinnamic acid		1.8	501-52-0	7	plant originated allelochemical [20, 21]
	cinnamic acid, p-methoxy		2.7	830-09-1	42	plant originated allelochemical [20, 21]
	4-hydroxybenzoic acid		1.6	99-96-7	8	plant originated allelochemical [20, 21]
	d-glucopyranose		-2.8	50-99-7	2	nectar [22]
	d-mannose		-3.4 [c]	3458-28-4	6	nectar [22]
	d-xylose		-2.7 [c]	58-86-6	2	nectar [22]
	d-glucose		-2.8	50-99-7	4	nectar [22]
	benzyl salicylate		4.3 [c]	118-58-1	2	plant originated allelochemical [20, 21]
	ferulic acid		1.5	1135-24-6	5	plant originated allelochemical [20, 21]
	caffeic acid (3,4-dihydroxy-cinnamic acid)		1.2	331-39-5	2	plant originated allelochemical [20, 21]
	benzyl cinnamate		3.4	103-41-3	6	plant originated allelochemical [20, 21]
	cinnamyl cinnamate		3.9	122-69-0	8	plant originated allelochemical [20, 21]
	chrysin		3.5	480-40-0	9	honey, propolis, and beeswax [23]
	stigmasterol (29δ (5,22))		9.4 [c]	83-48-7	9	pollen [24]
	beta-sitosterol (29δ (5))		9.7 [c]	83-46-5	29	pollen [24]
	lanosta-8,24-dien-3-ol, acetate, (3, beta)-		11.8 [c]	2671-68-3	6	pollen [24]

[a] alkenes identified by weight. the exact location of their double bonds are unknown. [b] alkenes with two double bonds [c] K_{ow} values estimated using the crippen method: episuite kowwin v1.67 estimate (usepa) (HSDB [1]).

Supplementary References

1. Hazardous Substances Data Bank (HSDB): A TOXNET Database. https://toxnet.nlm.nih.gov/newtoxnet/hsdb.htm (accessed 3/26/2019).
2. Dani, F.R.; Jones, G. R.; Corsi, S.; Beard, R.; Pradella, D.; Turillazzi, S. Nestmate Recognition Cues in the Honey Bee: Differential Importance of Cuticular Alkanes and Alkenes. *Chem. Senses* **2005**, *30 (6)*, 477-489.
3. Strachecka, A.; Borsuk, G.; Paleolog, J.; Olszewski, K.; Bajda, M.; Chobotow, J. Body-surface Compounds in Buckfast and Caucasian Honey Bee Workers (*Apis Mellifera*). *J. Apic. Sci.* **2014**, *58 (1)*, 5-15.
4. Kather, R.; Drijfhout, F. P.; Martin, S. J. Evidence for colony-specific differences in chemical mimicry in the parasitic mite *Varroa destructor*. *Chemoecology* **2015**, *25 (4)*, 215-222.
5. Murray, Z.L.; Keyzers, R. A.; Barbieri, R. F.; Digby, A. P.; Lester, P. J. Two pathogens change cuticular hydrocarbon profiles but neither elicit a social behavioural change in infected honey bees, *Apis mellifera* (Apidae: Hymenoptera). *Austral. Entomol.* **2016**, *55 (2)*, 147-153.
6. Okosun, O.O.; Yusuf, A.A.; Crewe, R.M.; Pirk, C.W.W. Effects of age and Reproductive Status on Tergal Gland Secretions in Queenless Honey bee Workers, Apis mellifera scutellata and A. m. capensis. *J. Chem. Ecol. [Online]* **2015**.
7. Trhlin, M., Rajchard, J. Chemical communication in the honeybee (*Apis mellifera L.*): a review. *Veterinarni Medicina* **2011**, *56 (6)*, 265-273.
8. Gilley, D.C. Hydrocarbons Emitted by Waggle-Dancing Honey Bees Increase Forager Recruitment by Stimulating Dancing. *PLoS ONE [Online]*. **2014**, *9*, e105671.
9. Coleman, R., Penner, D. Organic Acid Enhancement of Pelargonic Acid. *Weed Tech.* **2008**, *22 (1)*, 38-41.
10. Mărgăoan, R.; Mărghitaş, L.A.; Dezmirean, D.S.; Dulf, F.V.; Bunea, A.; Socaci, S.A.; Bobiş, O. Predominant and Secondary Pollen Botanical Origins Influence the Carotenoid and Fatty Acid Profile in Fresh Honeybee-Collected Pollen. *J. Agric. Food Chem.* **2014**, *62*, 6306-6316.
11. Zalewski, K.; Zaobidna, E.; Żółtowska, K. Fatty acid composition of the parasitic mite *Varroa destructor* and its host the worker prepupae of *Apis mellifera*. *Phys. Entom.* **2016**, *41*, 31-37.

12. Teerawanichpan, P.; Robertson, A.J.; Qiu, X. A fatty acyl-CoA reductase highly expressed in the head of honey bee (*Apis mellifera*) involves biosynthesis of a wide range of aliphatic fatty alcohols. *Insect Biochem. Mol. Biol.* **2010**, *40*, 641-649.
13. Buchwald, R.; Breed, M.D.; Bjostad, L.; Hibbard, B.E.; Greenberg, A.R. The role of fatty acids in the mechanical properties of beeswax. *Apidologie* **2009**, *40*, 585-594.
14. Wang, C. F.; Li, J. P.; Zhang, Y. B.; Zhang, Z. Z. Chemical constituents from the roots of Senecio scandens. *Chem. Nat. Comp.* **2011**, *47* (2), 243-245.
15. Ali, L.; Ahmad, R.; Rehman, N.U.; Khan, A.L.; Hassan, Z.; Rizvi, T.S.; Al-Harrasi, A.; Shinwari, Z.K.; Hussain, J. A New Cyclopropyl-Triterpenoid from *Ochradenus arabicus*. *Helvetica Chimica* Acta **2016**, *98*, 1240-1244.
16. Donzé, G.; Schnyder-Candrian, S.; Bogdanov, S.; Diehl, P-A.; Guerin, P. M.; Kilchenman, V.; Monachon, F. Aliphatic Alcohols and Aldehydes of the Honey Bee Cocoon Induce Arrestment Behavior in Varroa jacobsoni (Acari: Mesostigmata), an Ectoparasite of *Apis mellifera*. *Archives of Insect Biochemistry and Physiology*, **1998**, *37*, 129-145.
17. Terzo, M.; Urbanova, K.; Valerova, I.; Rasmont, P. Intra and interspecific variability of the cephalic labial glands' secretions in male bumblebees: the case of *Bombus (Thoracobombus) ruderarius* and *B. (Thoracobombus) sylvarum* [Hymenoptera, Apidae]. *Apidologie* **2005**, *36*, 85-96.
18. Mishra, P.K.; Sing, N.; Ahmad, G.; Dube, A.; Maurya, R. Glycolipids and other constituents from *Desmodium gangeticum* with antileishmanial and immunomodulatory activities. *Bioorg. Med. Chem. Letters* **2005**, *15 (20)*, 4543-4546.
19. Kamboj, A.; Pooja, A.; Saluja, A.K. Isolation and Characterization of Bioactive Compounds from the Petroleum Ether Extracts of Leaves of *Xanthium Strumarium Linn*. *BioMedRx* **2013**, *1 (3),* 235-238.
20. Yoneyama, K., Natsume, M. Phenolic Compounds. In *Comprehensive Natural Products II*; Mander, L., Liu, H-W. Eds,; Elsevier: Kidlington, UK, 2010; Volume 1, pp. 539-558.
21. Widhalm, J.R., Dudareva, N.A. A. Familiar Ring to It: Biosynthesis of Plant Benzoic Acids. *Mol. Plant*. **2015**, *8*, 83-97.
22. Nicolson, S. W. Bee Food: The Chemistry and Nutritional Value of Nectar, Pollen and Mixtures of the Two. *African Zool.* **2011**, *46 (2),* 197-204.
23. Vit, P.; Soler, C.; Tomás-Barberán, F. A. Profiles of phenolic compounds of *Apis mellifera* and *Melipona spp.* honeys from Venezuela. *Z. Lebensm. Unters. Forsch. A.* **1997**, *204*, 43-47.
24. Rasmont, P.; Regali, A.; Ings, T.C.; Lognay, G.; Baudart, E.; Marlier, M.; Delcarte, E.; Viville, P.; Marot, C.; Falmagne, P.; Verhaeghe, J-C.; Chittka, L. Analysis of Pollen and Nectar of *Arbutus unedo* as a Food Source for *Bombus terrestris* (Hymenoptera: Apidae). *J. Econ. Entomol*. **2005**, *98 (3),* 656-63.

© 2020 by the authors. Licensee MDPI, Basel, Switzerland. This article is an open access article distributed under the terms and conditions of the Creative Commons Attribution (CC BY) license (http://creativecommons.org/licenses/by/4.0/).
